# Comparing functional outcomes between 3D printed acetabular cups and traditional prosthetic implants in hip arthroplasty: a systematic review and meta analysis

**DOI:** 10.1007/s00402-024-05650-6

**Published:** 2024-12-27

**Authors:** Ryan St. John, Seth Spicer, Mo Hadaya, Hanna Brancaccio, Seungkyu Park, Sean McMillan

**Affiliations:** 1 Department of Medicine, Rowan-Virtua University School of Osteopathic Medicine, 1 Medical Center Dr, Stratford, NJ 08084 USA; 2 Futures Forward Research Institute, Toms River, NJ USA; 3 Virtua Health, Marlton, NJ USA

**Keywords:** 3D-printing, Acetabular cup, Hip, Arthroplasty

## Abstract

**Objective:**

The primary research aim was to determine if the use of traditional or 3D printed prosthesis resulted in better functional outcome scores in hip arthroplasty.

**Methods:**

A systematic review and meta-analysis was conducted utilizing the PRISMA 2020 guidelines. Six databases (PubMed, Embase, Scopus, WebOfScience, and Cochrane Library, Google Scholar) were searched yielding 1117 article titles and abstracts. Rayyan.ai was used to detect duplicates (n = 246) and for manual screening for inclusion and exclusion criteria. Included were controlled studies of any publication time that assessed Harris Hip Score (HHS) at baseline and twelve months. Six papers were sought for full text review of which three studies totaling 195 hips met final inclusion.

**Results:**

Mean HHS in the control group went from 38.15 (± 6.02) at baseline to 80.30 (± 4.79) at twelve months follow-up, while the 3D group saw a change from 37.81 (± 5.84) to 90.60 (± 4.49). Significant and large improvements between time points were seen within the control group [p = .02, Cohen’s d = 8.57 (1.48, 15.56)] and 3D group [p < 0.01, Cohen’s d = 9.18 (3.50, 14.86)]. The HHS score of the 3D group improved by 10.64 points more than the HHS score of the control group, which is a statistically insignificant (p = 0.89) amount.

**Conclusion:**

Group differences in pooled mean HHS scores at twelve months follow-up surpassed established minimum differences for clinical importance. High quality research should be further pursued to elucidate these findings.

## Introduction

Total hip arthroplasty (THA) is one of the most frequently performed surgeries worldwide [1]. The procedure has consistently been proven to result in clinically significant improvements in outcomes and high patient satisfaction [2]. Recent innovations in hip arthroplasty aim to extend the lifespan of the implant, improve functional outcomes, and reduce complications and revision rates [1, 3–5]. The success of these improvements stem from the ability to surgically recreate an individual’s positional anatomy and functional load bearing of the hip joint—the femoral head on the acetabular cup [6–10]. Other initiatives to enhance THA focus on patient expectations about postoperative quality of life and personalized joint replacement that can be tailored to each patient [11]. This can be accomplished through advancements in technology that enhance the precision of implantation, such as robotic navigation systems, and patient-specific instrumentation (PSI) [11].

Patient-reported outcome measures (PROMs) are an increasingly popular endpoint in clinical trials [12]. Validated PROMs ensure a standardized approach to assess participant perception of their outcomes, while providing unique information on the impact of a medical condition and patients’ experiences with treatment [13]. The Harris Hip Score (HHS) has been the most common PROM employed to assess the functionality of a patient with hip pathology before and after a surgical procedure [14, 15]. It is a patient and clinician report of pain, deformity, and range of motion [16]. The maximum score for the HHS is 100 signifying less dysfunction. A score of  < 70 is considered poor, 70–80 is considered fair, 80–90 is considered good, and 90–100 is considered excellent [14]. HHS has been shown to be the best clinician-based tool for the evaluation of hip arthroplasty success due to its high validity, reliability, and responsiveness compared to other PROMs.

Despite an increasing focus on the applicability of 3D-printing (3DP) technologies, there are currently no meta-analyses to determine the functional outcomes after receiving 3DP implants [17]. Research in 3DP prosthetic implants has demonstrated the technology’s capability to optimize implant-bone contact and filling of bone defects [18–20]. Mimicry of the biological extracellular bone matrix is thought to improve the osseointegration with the implant [21–25]. Additionally, 3DP cups are thought to better promote bone growth than traditionally manufactured cups due to increased porosity and homogeneity of the material [17]. In THA specifically, custom 3DP implants provide better rotational alignment and fit and are associated with lower blood loss, fewer adverse events, and decreased likelihood of discharge to a rehabilitation or acute care facility [26–28].

As a result of growing interest in 3DP applications for orthopedic applications and its significant potential to impact THA, we completed this systematic review and meta-analysis of current evidence to assess functional outcomes when implementing 3DP acetabular cups in THA.

## Methods

A systematic review and meta-analysis of Harris Hip scores (HHS) was conducted utilizing strict adherence to the Preferred Reporting Items for Systematic Reviews and Meta-Analyses (PRISMA 2020) guidelines (Page, 2020) [29]. This review was registered on PROSPERO and approved on January 21, 2024 ID: CRD42024501-001. The aim of this study was to determine if 3DP hip arthroplasty improved on clinical outcomes compared to non-3DP hip arthroplasty.

### Search procedure

A comprehensive and systematic review of several medical databases was conducted to search for articles investigating 3DP hip arthroplasty functional outcomes compared to non-3DP arthroplasty functional outcomes. On January 21st, 2024, a primary search of PubMed, Embase, Scopus, Web of Science, and Cochrane Library was conducted to retrieve all available articles with no limitations on the date of article publication. A secondary search of Google Scholar was performed on February 15th, 2024 which also had no limitations on the date of article publication.

MeSH was utilized to identify key terms which were subsequently applied to Boolean operators. The search string utilized for the primary search was (“3D” OR “three dimensional” OR “three dimension”) AND (“printed” OR “bioprint” OR “additive manufactured” OR “additive manufacturing”) AND (“Total Hip Replacements” OR “Total Hip Replacement” OR “Total Hip Arthroplasty” OR “Arthroplasty, Total Hip” OR “Hip Replacement Arthroplasties” OR “Hip Replacement Arthroplasty” OR “Hip Prosthesis Implantation” OR “Hip Prosthesis Implantations”). 531 retrieved studies were uploaded to Rayyan.ai after which duplicates were detected using the “Detect Duplicates” function within the platform. Two reviewers (RSJ and HB) then manually removed 246 articles and proceeded to confirm that no further duplicates were present. These same reviewers conducted title and abstract appraisal on the remaining 285 articles to determine which articles to further evaluate based on the predetermined inclusion and exclusion criteria.

**Fig. 1:**
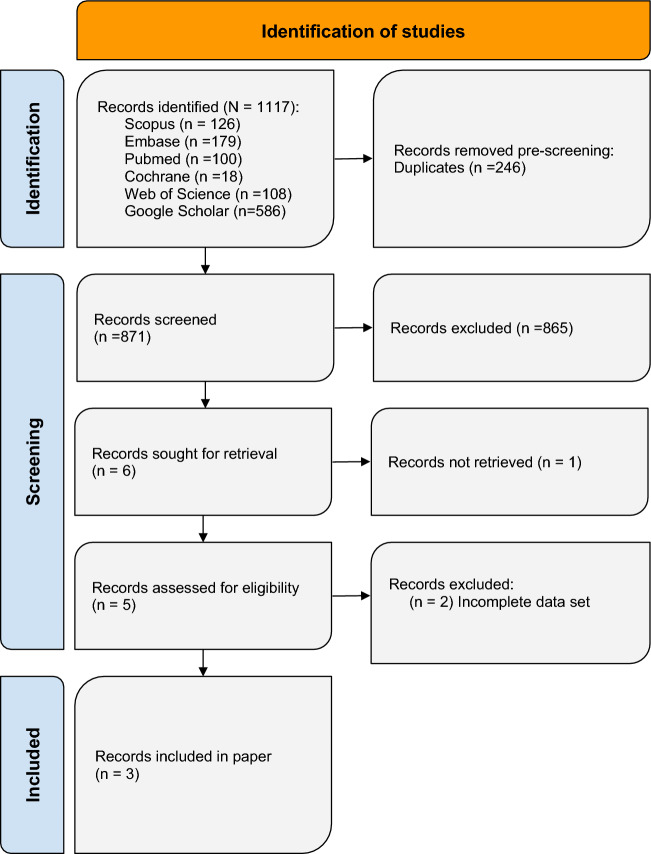
PRISMA flowchart illustrating the systematic review process. The flowchart outlines the selection and screening of studies, including search strategies, eligibility criteria, and the final inclusion of relevant studies for data synthesis and analysis

The search string utilized for the secondary search was (“3D printing” OR “3D printed” OR “3D bioprinting” OR “additive manufacturing” OR “additively manufactured”) AND (“total hip replacement” OR “total hip arthroplasty” OR “hip prosthesis implantation” OR “hip replacement arthroplasty”) AND (“Harris Hip Score” OR “HHS”). 586 studies were retrieved and manually screened by two reviewers (RSJ and HB) to determine relevance based on established inclusion and exclusion criteria (Fig. 1).

### Inclusion and exclusion criteria

Included articles were controlled studies that investigated 3DP hip components compared to non-3DP components. Articles also needed to have pre-procedure and post-procedure measurements of the HHS to determine the effects of the intervention on functionality. All included articles further required a post-procedure HHS measurement at twelve months. Excluded studies were articles that did not have a full text available and articles that did not have an English translation. Furthermore, case reports, case series, systematic reviews, and review articles were excluded. 6 articles were reviewed in their entirety before elimination due to incomplete data (n = 2) or because full text could not be retrieved (n = 1). For the remaining 3 articles, RSJ and SS extracted the relevant data to be used for statistical analysis (Table 1).

### Data collection and analysis

**Fig. 2:**
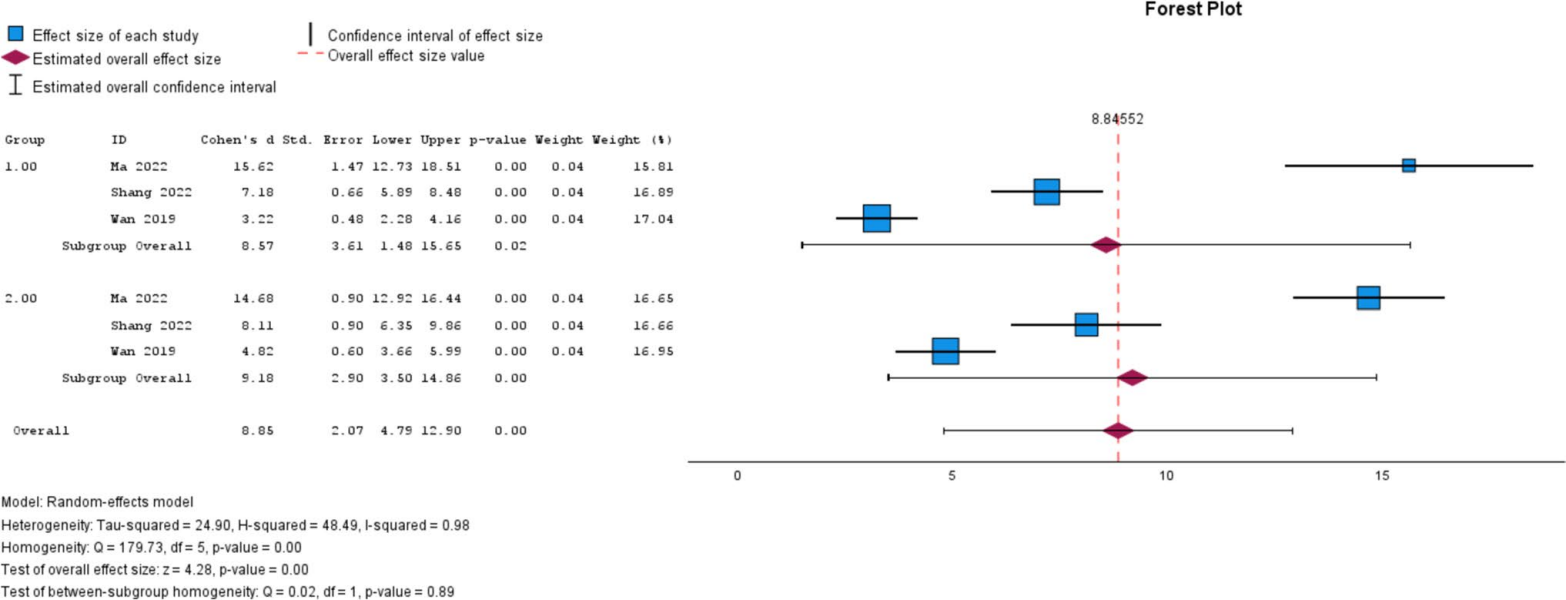
Forest Plots of the individual and pooled effect size, statistical significance, measures of heterogeneity, and measures of non-inferiority. Group 1 is control, Group 2 is 3D print group 33

Once study selection was complete, quantitative and qualitative data collection began. HHS was selected as the primary variable for our outcome study because it quantifies hip pain, function, and mobility. Some included studies reported multiple time points for follow-up evaluation of HHS, but all included studies shared follow-up evaluation at twelve months. Thus, mean, standard deviation, and sample size of the HHS data were collected at baseline and at twelve months follow-up. Weighted averages, standard deviations, and ranges of HHS scores were reported for both time points. Statistical analysis was performed using a meta-analysis with a random effects approach using IBM SPSS Statistics for Windows, version 29 (IBM Corp., Armonk, N.Y., USA) [30]. This approach pooled the effect sizes of individual studies, allowing for evaluation of the mean changes in HHS scores relative to variation. A p-value of less than 0.05 was utilized to determine statistical significance. The extent of improvement in HHS scores was represented by the pooled and individual effect size (Cohen’s D) with 95% confidence intervals [95% CI (LL, UL)]. Cohen’s D was used to represent the effect size (Fig. 2, Table2).

**Table 1:** Summary of evidence

Study id (first author, year)	Study design	Cup design (EXP v CTR)	# of patients (EXP v CTR)	AGE MEAN (EXP v CTR)	GENDER M/F (%) (3D v CTR)	HHS BASELINE MEAN (EXP v CTR)	HHS mean difference: baseline to 12 months (EXP v CTR)	Procedure indication
Ma, N. (2022)	Retrospective non-randomized control	3D Titanium alloy trabecular metal cup system (Beijing Aikang Yicheng Medical Equipment Co., LTD) v Pinnacle cup system (Johnson & Johnson Medical Device Co., LTD.)	96 [98 Hips] (69 v 29)	EXP: 60.66 ± 7.57 CTR: 59.48 ± 6.78	3D: 23.46% (M); 46.93% (F) CTR: 14.28% (M); 15.3% (F)	35.08 ± 4.74 v 34.19 ± 4.48	24.61 v 25.71	Femoral head necrosis: •EXP: 46 •CRT: 18 Osteoarthritis •EXP: 18 •CTR: 8 Hip Dysplasia •EXP: 5 •CTR: 3
Shang, G. (2022)	Retrospective non-randomized control	3D-printed TT acetabular cup (Aikang Corp., Beijing, China) v conventional porous titanium-coated acetabular cup (Reflection Acetabular System, Smith and Nephew, Memphis, TN, USA)	57 (23 v 34)	70.35 ± 8.10 v 71.62 ± 10.23	43.48% (M); 56.52% (F) CTR: 41.18% (M); 58.82% (F)	39.00 ± 6.16 v 37.77 ± 5.69	41.44 v 36.26	Aseptic Loosening: •EXP: 17 •CRT: 24 Infection: •EXP: 6 •CTR: 10
Wan, L. (2019)	Prospective non-randomized control	3D printed titanium trabecular cup v non-3D printed titanium alloy revision artificial hip joint	42 (22 v 20)	EXP:35.8 ± 6.7 CTR: 34.9 ± 5.9	3D: 54.55%(M); 45.45%(F) CTR: 55% (M); 45%(F)	45.11 ± 8.93 v 44.53 ± 8.83	43.46 v 27.62	N/A

Heterogeneity of the study results, representing variance, was primarily assessed using Q-statistics and the I^2^ ratio (I^2^ = τ^2^/H^2^) (Fig. 2). A test of subgroup homogeneity was used to represent differences between the control and 3DP groups, where statistical significance signifies that a difference is present. A larger I^2^ suggests more variance between study results, with observed differences potentially coming from another variable, such as bias in the study design. Tau-squared (τ^2^) represents the absolute variation between effect sizes, without considering the variation that is expected from random chance. A random effects model was utilized; this allows for analysis of variance greater than what would be expected from chance, thus delineating the influence of an external variable different from the influence of the dependent variable, whereas the less conservative common effects model does not. An H^2^ ratio was also reported to analyze variance, where a value of 1 represents equivalent variance between the two models. This would represent low heterogeneity as explained by no variation in study effect sizes greater than what is expected from random chance.

Descriptive analysis was used to determine the degree of osseointegration and cup stability following implantation. The degree of osseointegration was commonly assessed using the criteria of the Anderson Orthopaedic Institute which determines if appropriate osseointegration has occurred if at least three of the following are present: absence of radiolucent lines; presence of superolateral buttresses; presence of medial stress shielding; presence of radial trabeculae; and the presence of inferomedial buttresses. The stability of the cup was evaluated using the zonal analysis of DeLee and Charnley. This evaluates the width of radiolucent lines, changes in cup abduction angle, and distance of cup displacement. Using this method, loosening of the cup can be inferred if the abduction angle of the cup changes by more than 10 degrees or if there is cup migration of more than 6 mm in any direction. This same method determines adequate cup stability if radiolucent line width is less than 1 mm in two zones, no radiolucent lines in greater than two zones, and there is no cup displacement.

### Risk of bias and certainty of evidence assessment

**Fig. 3:**
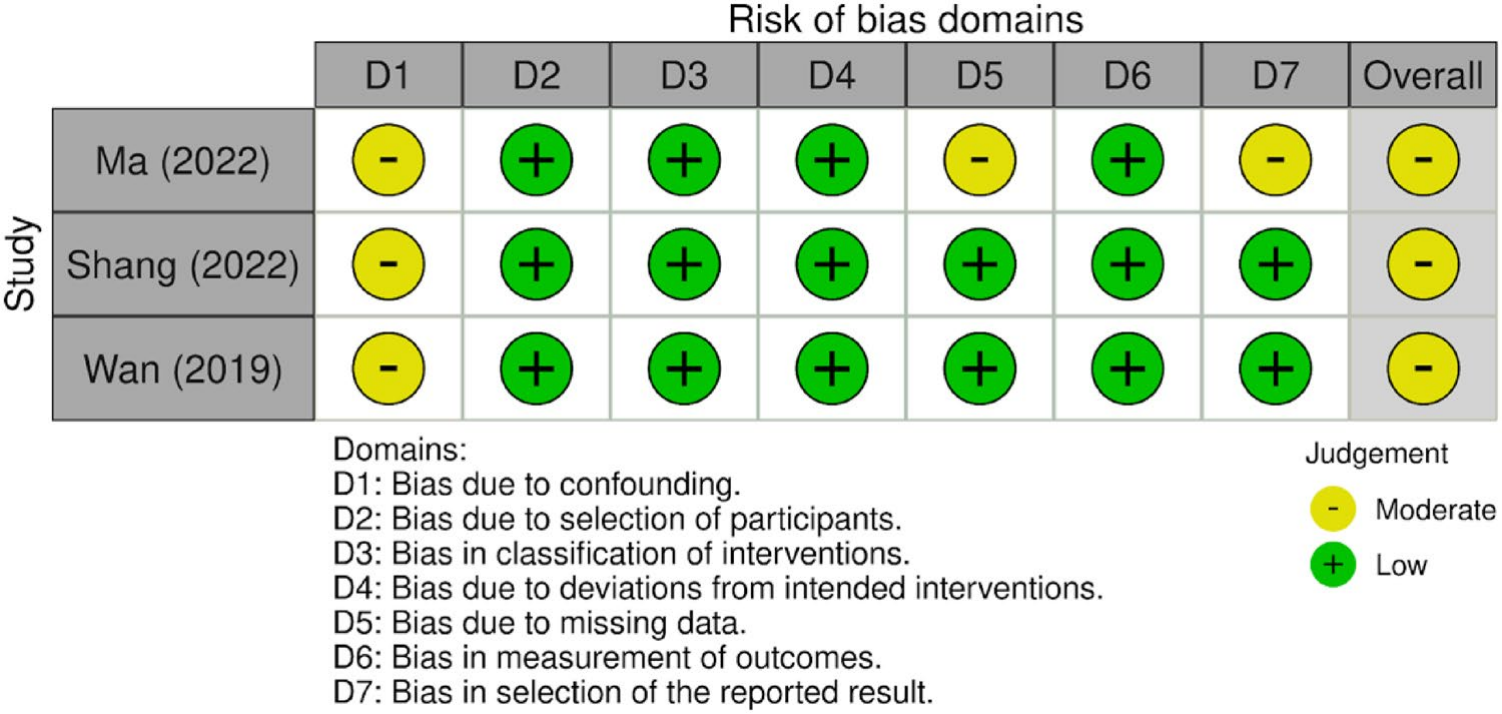
ROBINS-I stoplight plot (below) 33

**Fig. 4:**
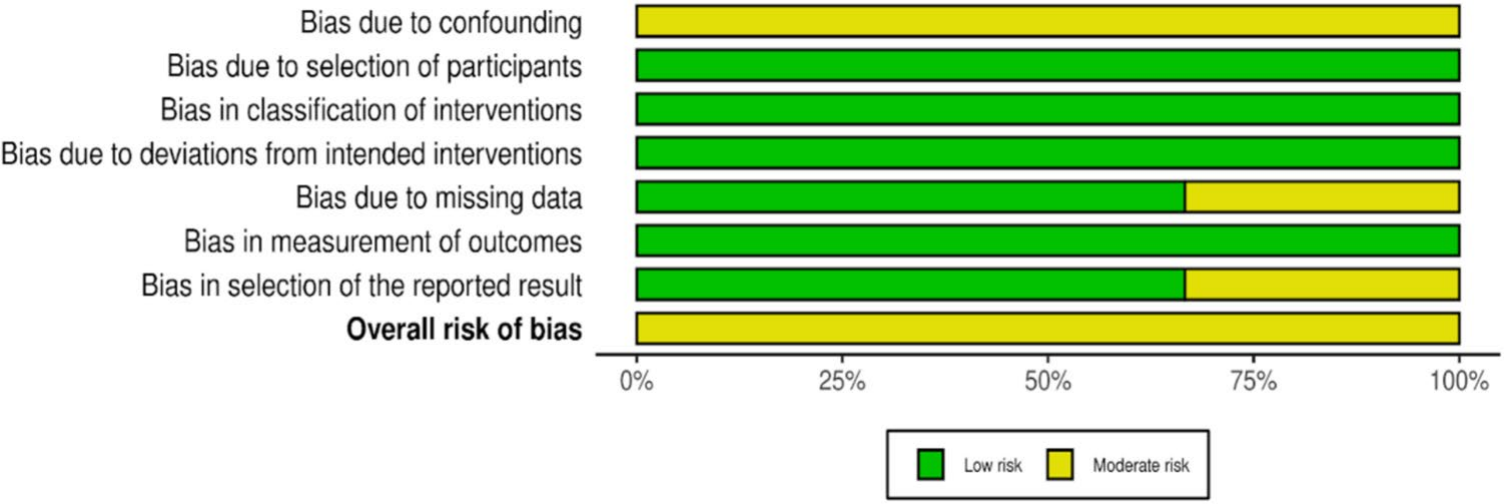
ROBINS-I summary of findings plot (below) [33]

Included manuscripts were evaluated for methodological quality using the Grading of Recommendations Assessment, Development and Evaluation (GRADE) method (Table 3) [31]. Bias in the included articles was also assessed independently by two authors (RSJ and HB) based on their respective study design. Cohort studies were subjected to evaluation with ROBINS-I and the data was presented in plot format (Figs. 3 and 4) [32].

**Table 2:** Data summary for the control and 3D groups

Control (CTR) vs 3D (3D Printed)	HIP CTR-12mo	HIP 3D-12mo
Number per group	83	114
Pooled average HHS Pre (± SD)	38.15 (± 6.02)	37.81 (± 5.84)
Pooled average HHS Post (± SD)	80.30 (± 4.79)	90.60 (± 4.49)
Average difference	42.15	52.79
Effect size [95% CI (LL, UL)]	8.57 [1.48, 15.56]	9.18 [3.50, 14.86]
Subgroup analysis of homogeneity (*p*-value)	0.89	0.89

### Included studies

Ma, M. et al. performed a retrospective non-randomized review on the short-term follow-up results of 3DP acetabular cups in total hip arthroplasty [34]. The control group had 29 hips using Pinnacle socket cups (Johnson and Johnson Medical Device Co., LTD.), while the observation group included 69 hips using 3DP titanium alloy bone trabecular socket cups (Beijing Aikang Yicheng Medical Equipment Co., LTD). Outcomes included pain visual analogue scale (VAS) scores, forgotten joint score, HHS, and imaging. These endpoints were measured preoperatively and at one, six, and twelve months postoperatively. Radiographic images were taken to observe implant stability and bone ingrowth as determined by the DeLee- Charnley criteria and the Kawamura radiological evaluation criteria.

Shang, G. et al. also conducted a retrospective non-randomized review on short and mid-term outcomes of a new 3DP trabecular titanium acetabular socket cup (Aikang Corp., Beijing, China) in comparison to a standard porous coated titanium acetabular cup (Reflection Acetabular System, Smith and Nephew, Memphis, TN, USA) [35]. The 3DP cups group consisted of 23 hips, and 34 hips received the porous coated titanium cup as a control. Recorded measures included VAS scores, Harris Hip Score, and Short Form 36. Anteroposterior hip joint radiographs were also acquired to assess limb-length discrepancy, bone ingrowth, cup stabilization, and upward movement of the hip center of rotation [36, 37]. Radiographs were taken preoperatively, one day postoperatively, and at the last follow-up. The other measurements were taken preoperatively, three months postoperatively, twelve months postoperatively, and at the last follow-up.

Lastly, Wan, L et al. utilized a prospective non-randomized controlled study design to explore the prognosis and effects of 3DP titanium alloy trabecular cups and pads in patients undergoing acetabular revision of the hip joint [38]. The control group non-3DP titanium trabecular cups and pads, while the experimental group utilized 3DP titanium alloy trabecular cups and pads. Pain VAS scores, HHS, and Quality of Life Health Survey (SF-36) scores were recorded and compared between the groups. Measurements were taken preoperatively as well as three months postoperatively, six months postoperatively, and twelve months postoperatively. Imaging was done to assess acetabular prosthesis position and bone ingrowth. Acetabular prosthesis position was assessed with the zonal analysis of DeLee and Charnley [36]. Bone ingrowth followed criteria by the Anderson Orthopedic Research Institute. Images were taken at follow-ups, although the study only mentions results one week postoperatively, six months post-operatively, and at the 12 month follow-up.

## Results

### Baseline characteristics

Three studies were included comparing highly porous 3DP acetabular cups compared to non-3DP acetabular cups with conventional porosity. The number of participants in each study, as well as age, gender and baseline HHS can be found in Table 1. Patient age varied, with the majority of participants being 60 years of age or older. One study had a lower mean participant age compared to the other studies. All participants in the included studies had a mean HHS in the “very poor” range at baseline. Two of the included studies provided indications for the procedures which included femoral head necrosis, osteoarthritis, hip dysplasia, aseptic loosening and infection (Table 1). All of the 3DP acetabular cups used in the experimental group were highly porous, trabecular metal, hemispheric cups with pore sizes ranging from 600 to 1000 μm. Control group acetabular cups were also hemispheric cups with pore sizes ranging from 100 to 400 μm.

### Effect of intervention

HHS substantially improved in the control group and 3DP group in each of the three studies. The pooled mean HHS value for the control group was 38.15 (± 6.02) at baseline and 80.30 (± 4.79) at twelve months follow-up, an improvement of 42.15. Within the 3DP group, HHS value was 37.81 (± 5.84) at baseline and 90.60 (± 4.49) at twelve months follow-up, an improvement of 52.79 (Table 2). Improvement was seen between baseline and twelve months follow-up in the control group [p = 0.02, Cohen’s D = 8.57 (1.48, 15.56)] and the 3D group [p < 0.01, Cohen’s D = 9.18 (3.50, 14.86)]; however, subgroup analysis of homogeneity revealed no significant differences between groups (p = 0.89) (Fig. 2).

### Heterogeneity

In the control group, measures of heterogeneity were found to be I^2^ = 98.6%, τ^2^ = 38.2, and H^2^ = 71.0. In the 3DP group, I^2^ = 97.5%, τ^2^ = 24.6, and H^2^ = 40.5. The design variables, which may have affected the variance in results between studies, were examined in the risk of bias and quality of evidence assessments.

### Radiographical findings

According to the zonal analysis of DeLee and Charnley, none of the cups in the 3DP groups displayed migration or changes in abduction angle throughout the follow-up period signifying stable fixation. Of note, two patients in the 3DP group did have radiolucent lines around the cup one week after surgery however these lines disappeared by 6 months. Similarly, non-3DP cups also showed adequate fixation with no evidence of instability. Four patients did demonstrate radiolucent lines at one week, however the lines disappeared by 6 months as well.

Osseointegration was determined by the criteria defined by the Anderson Orthopedic Research Institute. In the studies by Ma, N. et al., and Wan L. et al., all patients demonstrated a well-fixed acetabular component. However, Shang, G. et al. demonstrated a significant difference in bone ingrowth between the two groups with the 3DP having a statistically significant higher rate of osseointegration (p = 0.037). In this study, only two patients in the 3DP group demonstrated less than three of the signs which are needed to signify adequate bone ingrowth. However, in the control group, ten of the non-3DP cups showed less than three of the necessary radiographical signs. Overall, this study demonstrated that rate of bone ingrowth for the 3DP and control groups was 91.3% and 70.59% respectively.

### Risk of bias and certainty of evidence assessment

GRADE analysis revealed a moderate certainty of evidence for this study (Table 3). Two studies were retrospective non-randomized controlled studies while the third was a prospective nonrandomized study. The lack of randomization contributes to an inherent risk of bias in these studies. Due to the heterogeneity between studies, reviewers determined there to be a serious risk of inconsistency; however, we are unable to determine its significance. Other factors that are taken into account for upgrading quality of evidence (i.e., large effect size, dose–response gradient, and plausible confounders that would have reduced effect size) were not applicable.

**Table 3:** GRADE chart

Participants (Studies)	Risk of Bias	Inconsistency	Indirectness	Imprecision	Other Considerations	Overall certainty of evidence
Does the use of 3D printed prosthesis result in better functional outcomes following hip arthroplasty?						
195 (2 retrospective non-randomized controlled studies, 1 prospective review)	Not serious	Serious	Not serious	Not serious	Undetected	Moderate
Explanation		High heterogeneity				

The Risk of Bias assessment revealed some concerns in the three included studies. All three studies were found to have some concerns with risk of bias due to confounders. One study was found to have moderate risk of bias due to missing data. One study was found to have moderate risk of bias due to selection of the reported result. However, the risk of bias across these two domains was not enough to substantially lower confidence in the results of the study (Figs. 3 and 4).

## Discussion

The goal of this meta-analysis was to assess if 3DP prosthetics improves patient outcomes compared to conventional approaches. This is the first meta-analysis to quantify functional outcomes associated with this procedural technique. The retrospective cohorts reviewed provide a fresh perspective by comparing the clinical outcomes of 3DP intervention and conventional treatment in similar patient populations In the present study, the 3DP group HHS did not achieve a statistically significant increase in effect; however, since HHS scored increased greater than 10 points (10.64) relative to control, 3DP acetabular cups did achieve a significantly higher minimal clinically important difference compared to traditional acetabular cups. Given the high heterogeneity (I^2^ = 98.6% in Group 1, 97.5% in Group 2) and small number of studies, these results provide moderate support to the hypothesis that 3DP implants result in better functional outcomes. This highlights how higher quality evidence in the form of randomized clinical trials are needed to corroborate these findings.

3DP implants have a few significant advantages in adaptability and mechanical properties that may have contributed to the observed improvement in functional outcomes. It is unlikely that comorbidities played a role in the observed differences due to the lack statistically significant differences between patient populations in each respective study. The high porosity of 3DP implants may offer better osseointegration and stability; this is consistent with the comparatively higher levels of bone in growth and stability in the 3DP groups [39, 40]. Shang G. et al.’s study found similar results, and the 3D group had statistically significant improvements of bone ingrowth compared to control (p = 0.037) [35]. The radiographic outcomes in both the Ma et al. study and Wan et al. study showed good stability and grow-in ability in both the 3DP and standard implant group [34, 38]. 3DP implants can also have their shape adapted to each patient’s specific anatomy, and 3DP joints may have a significant advantage in cases where bone is irregularly shaped [41]. This is especially important in reducing revisions for hip arthroplasty, as irregularly shaped bone is a significant risk factor [42]. If the observed results in the 3DP group are due to improved osseointegration and a more stable construct, they may also reduce the rate of revisions, significantly lowering costs and recovery time [43]. Regarding 3DP acetabular cups, which were used in all the studies, they are thinner, less expensive, and have a wider variety of diameter of femoral heads and shells [44]. This could contribute to a better fit and consequently, better outcomes. Further research utilizing the adaptability and flexibility of 3DP acetabular cups could improve outcomes and reduce stress on neighboring joints in some cases. Different materials and structures are also being explored in the context of 3DP stems, such as those which use tuned porous architecture to simulate the natural stiffness of bone and consequently reduce stress shielding [45].

There were a few limitations in this meta-analysis. All studies on 3DP hip arthroplasty were limited in scope, observing the outcome of 3DP acetabular cups in revision arthroplasty. Given that revision arthroplasty generally has worse outcomes than primary arthroplasty, research is needed to assess if the benefits of 3DP arthroplasty can be extrapolated to primary arthroplasty. Another point of note in our studies is that cohort studies are prone to confounding variables and limited in establishing causation. Conducting randomized controlled trials would strengthen the hypotheses on the impact of 3DP acetabular cups. The other main point of improvement is in outcome assessment. 3DP currently shows promise in reducing operation time and improving outcomes in patients needing hip arthroplasty, such as by providing models for preoperative planning and patient-specific guides [46, 47]. A retrospective analysis on the usage of 3D printing technology in acetabular fracture fixation found that 3DP patient pre-contoured plates and computer-assisted virtual surgical procedures resulted in improved operative time and reduced intraoperative blood loss [48]. This analysis compared HHS scores, which were utilized by all the studies. While HHS is frequently used, operation time and tests reliant on patient input such as Hip Disability and Osteoarthritis (HOOS) scores and Oxford Hip scores may provide different perspectives. Lastly, there are differences in design between manufacturers that may influence clinical outcomes [49].

## Conclusion

Based on current studies, 3DP hip arthroplasty shows promise in improving hip outcomes compared to conventional treatment in the short term. However, given the limited data and lack of randomized controlled trials in the field and current studies being limited to outcomes at up to 12 months, more research is needed to confirm this hypothesis and the long term utility of 3DP acetabular cups. If findings are positive, the adaptability and flexibility of 3DP acetabular cups and joints could reduce complications, improve recovery time, and potentially be applied to other joint replacement surgery.

## Data Availability

The data that support the findings of this study are available from the corresponding author, [RS], upon reasonable request.

## Abbreviations

HSS: Harris hip score
THA: Total hip arthroplasty
3DP: 3D-printing
PSI: Patient-specific instrumentation
